# Oncology drug pricing: potential Medicare savings on cancer-directed and supportive care medications through the Mark Cuban cost plus drug model

**DOI:** 10.1093/oncolo/oyae083

**Published:** 2024-05-13

**Authors:** Max J Bouvette, Anh B Lam, Christopher Bouvette, Adanma Ayanambakkam, Ryan D Nipp

**Affiliations:** College of Medicine, University of Oklahoma Health Sciences Center, Oklahoma City, OK, United Ststes; Department of Medicine, University of Oklahoma Health Sciences Center, Oklahoma City, OK, United States; Department of Medicine, University of Oklahoma Health Sciences Center, Oklahoma City, OK, United States; Section of Hematology/Oncology, Department of Medicine, Stephenson Cancer Center and the University of Oklahoma Health Sciences Center, Oklahoma City, OK, United States; Section of Hematology/Oncology, Department of Medicine, Stephenson Cancer Center and the University of Oklahoma Health Sciences Center, Oklahoma City, OK, United States

**Keywords:** health care costs, prescription drugs, Medicare, negotiations, financial toxicity, policy

## Abstract

Prescription drug costs within oncology remain a challenge for many patients with cancer. The Mark Cuban Cost Plus Drug Company (MCCPDC) launched in 2022, aiming to provide transparently priced medications at reduced costs. In this study, we sought to describe the potential impact of MCCPDC on Medicare Part-D oncology spending related to cancer-directed (*n* = 7) and supportive care (*n* = 26) drugs. We extracted data for drug-specific Part-D claims and spending for 2021. Using 90-count purchases from MCCPDC, we found potential Part-D savings of $857.8 million (91% savings) across the 7 cancer-directed drugs and $28.7 million (67% savings) across 21/26 (5/26 did not demonstrate savings) supportive care drugs. Collectively, our findings support that alternative purchasing models like MCCPDC may promote substantial health care savings.

## Teaser

Prescription drug costs are a challenge for patients with cancer. The Mark Cuban Cost Plus Drug Company (MCCPDC) launched in 2022, aiming to provide transparently priced medications at reduced costs. This article describes the potential impact of MCCPDC on Medicare spending within oncology.

## Introduction

Exorbitant prescription medication costs contribute to patients experiencing financial toxicity, defined as the financial burden of health care costs impacting patients’ care, quality of life, and clinical outcomes.^1-3^ Medicare Part-D provides beneficiaries with prescription coverage, but this program has historically faced legislative barriers to drug price negotiations.^4^ In 2022, Mark Cuban launched the Mark Cuban Cost Plus Drug Company (MCCPDC), a direct-to-consumer model seeking to provide prescription drugs to patients at reduced prices.^5^ MCCPDC negotiates directly with drug manufacturers for competitive acquisition prices and offers prescriptions at wholesale cost plus 15% markup, along with transparent pharmacy and shipping fees.^5^ Prior literature demonstrated the potential advantage of Medicare purchasing at MCCPDC price points across several cancer-directed therapeutics.^6^ Here, we aim to build upon these findings through cost analysis of cancer-directed and supportive care drugs used within oncology, further illustrating the potential impact of MCCPDC on Medicare Part-D spending.

## Materials and methods

Oncology claims were extracted from 2021 (most recently available) Part-D claims data accessible via Centers for Medicare and Medicaid Services. To identify common cancer-directed and supportive care drugs, we ranked medications according to claim volume. Drugs with <1000 claims were excluded. Of the 249 remaining drugs, roughly half were available for purchase through MCCPDC as of June 2023 (123/249, 49%). These were categorized into 3 groups: (1) cancer-directed (*n* = 8), (2) supportive care (*n* = 35), and (3) noncancer directed (*n* = 80). Noncancer-directed drugs were excluded. Ten drugs were also excluded due to poor differentiation of formulation or administration route. The final sample comprised 33 drugs (*n* = 7 cancer-directed, *n* = 26 supportive care).

We assessed claims for cancer-directed drugs with the Part-D Spending by Drug dataset, acknowledging that some drugs may have limited nononcology applications. Supportive care medications are prescribed across medical care for a variety of conditions, and thus we restricted savings estimation on this category to cancer-specific claims rather than all of Medicare. We obtained the units/claim from Part-D Spending by Drug data and multiplied by the total cancer-specific claims within the Part-D Provider and Drug data to estimate dosage units specific to oncology.

We performed a cost analysis to estimate Medicare spending if prescriptions were purchased through MCCPDC at supply-specific price points. Estimates included MCCPDC shipping and pharmacy fees. Actual spending for 2021 Medicare was compared against cost projection using MCCPDC 30-count (30c) and 90-count (90c) supplies. When MCCPDC offered multiple dosages, a commonly prescribed option was selected to prevent savings overestimation. The National Average Drug Acquisition Cost (NADAC) database was used to account for cost changes in manufacturing between 2021 and 2023, allowing for estimations of Medicare savings at present day, 2023 US dollars.

## Results

Total Part-D spending for 2021 was $216 billion (2021 USD). Across the cancer-directed (*n* = 7) drug cohort, 2021 Medicare spending totaled $1.3 billion. NADAC-adjusted Medicare spending was $947.5 million (2023 U.S. dollars). Potential savings if Medicare purchased MCCPDC 30c supplies totaled $806.6 million (85% savings) across 6/7 drugs. Only anastrozole demonstrated a disadvantaged MCCPDC 30c price point compared to Medicare (+7%). No cancer-directed drugs demonstrated a disadvantaged MCCDPC 90c price point. Potential savings if Medicare purchased MCCPDC 90c supplies totaled $857.8 million (91% savings) across 7/7 drugs. The top 5 drugs by estimated 90c savings were: abiraterone ($524.2 million; 96% savings), imatinib ($241.9 million; 97% savings), methotrexate ($37.5 million; 66% savings), erlotinib ($19.7 million; 97% savings), and anastrozole ($14.9 million; 40% savings; Table 1).

**Table 1.** Cancer-directed drugs—potential Medicare savings with MCCPDC 30C and 90C purchasing.

Medication ranking by Medicare claim volume	Generic drug name	Estimated Medicare savings at MCCPDC 30C pricing (2023 USD)	Percent savings w MCCPDC 30C	Estimated Medicare savings at MCCPDC 90C pricing (2023 USD)	Percent savings w MCCPDC 90C
1	Anastrozole	−$2 743 813.96	−7%	$14 927 185.99	40%
2	Letrozole	$4 495 247.72	21%	$13 111 760.93	60%
10	Tamoxifen citrate	$1 902 497.07	14%	$6 556 799.92	47%
19	Abiraterone acetate	$520 217 617.57	95%	$524 155 167.97	96%
30	Imatinib mesylate	$240 870 258.65	97%	$241 868 199.54	97%
155	Erlotinib HCl	$19 689 307.21	97%	$19 744 484.63	97%
164	Methotrexate sodium	$19 405 220.49	34%	$37 473 605.19	66%

Across the supportive care (*n* = 26) drugs, Medicare spending totaled $46.7 million. NADAC-adjusted Medicare spending was $42.8 million (2023 US dollars). Potential savings if Medicare purchased MCCPDC 30c supplies totaled $24.5 million (57% savings) across 10/26 drugs. Sixteen drugs (16/26; 62%) had a disadvantaged MCCPDC 30c price point. Potential savings if Medicare purchased MCCPDC 90c supplies totaled $28.7 million (67% savings) across 21/26 drugs. The top 5 drugs by estimated 90c savings were: deferasirox ($21.8 million; 98% savings), ondansetron HCl ($2.9 million, 61% savings), ondansetron ODT ($1.3 million; 61% savings), pantoprazole ($452,000; 35% savings), and duloxetine ($402,000; 58% savings; Table 2). Only 5 (5/26) supportive care drugs demonstrated a disadvantaged MCCPDC 90c price point: naproxen (+2%), meloxicam (+6%), hydroxyzine (+16%), dexamethasone (+34%), and metoclopramide (+69%).

**Table 2.** Supportive care drugs—potential Medicare savings with MCCPDC 30C and 90C purchasing.

Medication ranking by Medicare claim volume	Generic drug name	Estimated Medicare savings at MCCPDC 30C pricing (2023 USD)	Percent savings w MCCPDC 30C	Estimated Medicare savings at MCCPDC 90C pricing (2023 USD)	Percent savings w MCCPDC 90C
3	Prednisone	−$2 201 502	−120%	$60 166	3%
5	Dexamethasone	−$3 908 678	−81%	−$1 641 523	−34%
6	Ondansetron HCl	$1 199 494	25%	$2 875 840	61%
22	Ondansetron ODT	$738 762	36%	$1 253 439	61%
25	Pantoprazole sodium	−$611 741	−48%	$451 619	35%
28	Omeprazole	−$818 073	−89%	$159 119	17%
50	Venlafaxine HCl	−$56 619	−9%	$352 606	57%
51	Mirtazapine	$107 098	23%	$311 160	66%
52	Famotidine	−$343 723	−119%	$35 733	12%
53	Duloxetine HCl	$82 127	12%	$401 842	58%
54	Promethazine HCl	−$235 248	−83%	$74 966	27%
75	Methylprednisolone	−$10 504	−8%	$42 718	33%
82	Loperamide HCl	−$100 218	−40%	$56 307	23%
107	Metoclopramide HCl	−$164 101	−303%	−$37 587	−69%
114	Meloxicam	−$59 787	−166%	−$2331	−6%
117	Cyclobenzaprine HCl	−$24 426	−30%	$38 838	48%
120	Deferasirox	$21 758 071	98%	$21 831 575	98%
123	Ibuprofen	−$57 749	−122%	$5336	11%
124	Celecoxib	$71 995	40%	$131 603	74%
144	Hydroxyzine HCl	−$57 525	−131%	−$7038	−16%
150	Esomeprazole Mg	$129 053	63%	$166 463	81%
154	Aprepitant	$388 500	30%	$391 542	30%
166	Baclofen	−$24 775	−49%	$18 714	37%
177	Quetiapine fumarate	$575	1%	$21 624	48%
209	Granisetron HCl	$56 762	52%	$65 258	60%
219	Naproxen	−$16 979	−123%	−$213	−2%

## Discussion

We demonstrated the potential for MCCPDC to provide substantial Medicare cost savings on cancer-directed and supportive care medications within oncology. We identified drugs currently offered by MCCPDC with advantaged price points, underscoring opportunities to facilitate cost-conscious care and help guide patient-clinician conversations about prescription drug costs. This may assist efforts to mitigate financial toxicity in oncology. Prescription purchasing through MCCPDC, specifically at 90-count pricing, showed advantageous pricing across most drugs in our sample (28/33 [85%]). These results may reflect prior successful price negotiation efforts between MCCPDC and manufacturers while also highlighting the feasibility and potential for such negotiations.

Previous studies across varied fields have described potential Medicare savings with MCCPDC; however, the current study represents the first to report cost analysis of both cancer-directed and supportive care medications used in oncology.^6-8^ Although our findings may vary slightly from those of Cortese et al,^6^ these variations could be attributed to MCCPDC price changes over time, along with differences in methodology such as data sources, dosage choices, and changes within the public market over time. This study has limitations that merit discussion. Findings are restricted solely to Medicare claims on 33 drugs and likely underestimate the broader financial impact of MCCPDC, particularly among the uninsured.

Notably, our results do not communicate direct, out-of-pocket savings to patients but rather an overall reduction in Medicare Part-D spending. Additionally, we used Part-D data to estimate the number of dosage units prescribed specifically within oncology, and this may not perfectly estimate the exact amounts. Projections of potential savings are limited, as future Medicare spending patterns may change with the Inflation Reduction Act. Cost erosion on generic drugs may also occur after loss of exclusivity.^9^ Moreover, hurdles to the implementation of alternative drug sources on a larger scale may exist. Future efforts should seek to conduct prospective studies assessing the direct impact of MCCPDC on patients’ out-of-pocket costs and patient-reported outcomes.

## Conclusion

In this study, we demonstrated cost savings on cancer-directed and supportive care drugs used within oncology, further illustrating the potential impact of MCCPDC on Medicare Part-D spending.

## Data Availability

Three datasets were used in this cost analysis, all of which are freely accessible and compatible with Microsoft Excel software. The Medicare Part-D Spending by Drug 2021 and Medicare Part-D Prescribers by Provider and Drug 2021 (Filtered by Medical Oncology/Hematology-Oncology) datasets are both open access via the Centers for Medicare & Medicaid Services website, specifically under the subtopics of “Summary Statistics on Use and Payment” and “Provider Summary by Type of Service.” The National Average Drug Acquisition Cost dataset is also freely available via the US Centers for Medicare & Medicaid Services website. These datasets are linked below, and specific variables are described within text. This study was not submitted for IRB review due to its sole use of publicly available, de-identified data that did not meet human subject research (https://data.cms.gov/summary-statistics-on-use-and-payments/medicare-medicaid-spending-by-drug/medicare-part-d-spending-by-drug; https://data.cms.gov/provider-summary-by-type-of-service/medicare-part-d-prescribers/medicare-part-d-prescribers-by-provider-and-drug; https://data.medicaid.gov/nadac).
